# Nb-Doped g‑C_3_N_4_: A Highly Efficient and Stable Catalyst for Oxidative Desulfurization

**DOI:** 10.1021/acsomega.6c05450

**Published:** 2026-10-20

**Authors:** Jennefer V. M. Oliveira, Jhonatan B. Oliveira, Klaus Krambrock, Breno R. L. Galvao, Raquel V. Mambrini, Eudes Lorençon

**Affiliations:** † 74355 Centro Federal de Educação Tecnológica de Minas Gerais (CEFET-MG), Departamento de Química, Av. Amazonas 5253, Nova Suíça, Belo Horizonte, Minas Gerais 30421-169, Brazil; ‡ 28114 Universidade Federal de Minas Gerais (UFMG), Departamento de Física, Av. Antônio Carlos 6627, Pampulha, Belo Horizonte, Minas Gerais 31270-901, Brazil

## Abstract

This study reports the synthesis and application of niobium-doped graphitic carbon nitride as a high-performance catalyst for oxidative desulfurization (ODS) of fuels. The 10-NbCN material, prepared via pyrolysis, exhibits Nb species that are highly dispersed at the atomic level within the g-C_3_N_4_ framework, as confirmed by XRD, FTIR, and TEM. Under optimized conditions (60 °C, O/S = 20:1, 4 g L^–1^ catalyst), 96% removal of dibenzothiophene (DBT) is achieved within 120 min, outperforming pure g-C_3_N_4_ and Nb_2_O_5_. Kinetic studies reveal pseudo-first-order behavior with an activation energy of 41.2 kJ mol^–1^. Remarkably, quinoline enhances DBT conversion even at high concentrations, indicating an unusual tolerance to nitrogen-containing compounds typically known to inhibit ODS systems. Evaluation in real diesel confirms that 10-NbCN selectively removes sulfur compounds while preserving the hydrocarbon matrix. EPR and DFT results suggest that Nb sites efficiently activate H_2_O_2_, promoting the generation of reactive oxygen species. The catalyst demonstrates outstanding stability over six cycles with negligible Nb leaching, supported by the preservation of Nb–N coordination under oxidative conditions. These findings highlight Nb–g-C_3_N_4_ as a robust and N-tolerant catalyst for ultralow sulfur fuel production.

## Graphical abstract

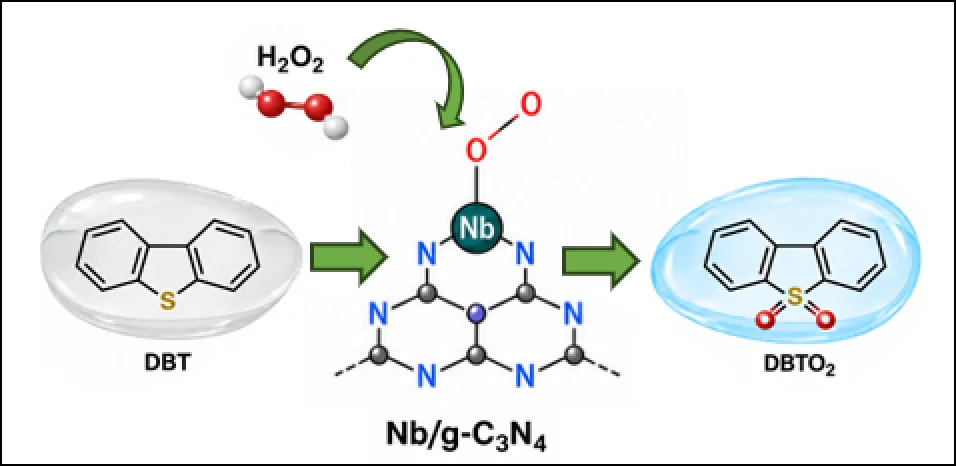

## 1 Introduction

The increasing environmental concerns and stringent regulations on sulfur emissions have necessitated the development of advanced and sustainable fuel desulfurization technologies.^1,2^ Oxidative desulfurization (ODS) has emerged as a promising alternative to conventional hydrodesulfurization (HDS), offering the ability to remove refractory sulfur compounds under mild operating conditions.^1,3^ Unlike HDS, which requires high temperatures and pressures, ODS utilizes oxidants and catalysts to convert sulfur compounds into sulfones or sulfoxides, which can be readily separated from fuels using polar solvents or adsorbents.^4,5^ This approach is particularly effective for achieving ultralow sulfur levels in fuels, thereby addressing the limitations of HDS in treating complex sulfur species such as dibenzothiophene (DBT).^5^ The performance of ODS processes is heavily dependent on the choice of catalytic materials and optimization of reaction parameters, such as oxidant concentration and catalyst dosage.^6,7^ Beyond catalytic oxidation, other non-hydrogen desulfurization routes include extractive, adsorptive, and biological processes. Bioconversion is particularly attractive because oxidized sulfur products can be further treated rather than regarded only as waste. Benzo- and dibenzothiophene sulfones have been anaerobically converted into inorganic sulfide with simultaneous biogas production, while sulfur-containing extracts obtained from H_2_O_2_-preoxidized petroleum streams have also been processed using immobilized anaerobic consortia.^8,9^ Peroxide-based ODS has likewise been demonstrated for both model fuels using reusable molybdenum-containing catalysts and complex petroleum feedstocks, including nonhydrotreated vacuum gas oil/diesel mixtures.^10,11^ These studies highlight the potential integration of mild chemical oxidation with separation and subsequent biological conversion of sulfur-rich products.

In recent years, significant attention has been directed toward developing innovative catalytic materials, particularly graphitic carbon nitride (g-C_3_N_4_) and its derivatives, for applications in catalysis and photocatalysis.^12,13^ g-C_3_N_4_ is a semiconductor with a bandgap of approximately 2.7 eV, enabling efficient absorption of visible light. Its straightforward synthesis through the thermal polycondensation of nitrogen-rich precursors, such as urea, melamine, and Dicyandiamide, makes it attractive for various photocatalytic processes, including pollutant degradation, water splitting, and CO_2_ reduction.^12–14^ Moreover, the pyridinic nitrogen atoms in g-C_3_N_4_ provide coordination sites for the incorporation and stabilization of transition-metal ions via complexation or chelation.^15^ This enables g-C_3_N_4_ to act as a platform for creating catalysts with well-dispersed and durable metal sites, maximizing the use of catalytically active metals and thus improving stability and performance.^16^ For instance, Zhou et al. synthesized Co-doped g-C_3_N_4_, which demonstrated excellent catalytic performance in the removal of ciprofloxacin using peroxymonosulfate as an oxidant.^17^ Similarly, Fe-doped g-C_3_N_4_ has been prepared via thermal polymerization and employed as a Fenton-like catalyst.^18,19^ Jiang et al.^20^ successfully synthesized highly dispersed Fe-doped g-C_3_N_4_ through a simple copolymerization strategy. The resulting catalysts exhibited excellent activity for the oxidation of H_2_S to elemental sulfur while demonstrating remarkable resistance to water and sulfur poisoning, ensuring exceptional catalytic stability.

In parallel, Niobium-based materials have also gained attention for their catalytic properties in oxidation reactions involving hydrogen peroxide.^21–23^ For example, Zhang et al.^22^ demonstrated the effectiveness of amorphous Nb_2_O_5_ supported on activated carbon for the selective oxidation of sulfide (methyl phenyl sulfide) to sulfone using hydrogen peroxide, while Wang and collaborators highlighted the ability of niobium oxyhydroxide and niobium oxide to activate hydrogen peroxide for the oxidation of dimethyl sulfoxide to dimethyl sulfone.^24^ Wolski et al. reported that niobium pentoxide (Nb_2_O_5_) can effectively degrade methylene blue with H_2_O_2_.^21^ However, a significant drawback of niobium-based catalysts is their inherently low surface area and the leaching of active Nb sites in the form of peroxo-niobate species when exposed to H_2_O_2_, compromising their long-term stability and catalytic efficiency.^21,22^ Wang et al. report that the pristine 20% Nb_2_O_5_/SiO_2_ catalyst exhibits exceptional catalytic activity in the oxidation of dimethyl sulfoxide to the corresponding sulfone using H_2_O_2_.^25–27^ However, its reusability is limited due to the leaching of Nb species during consecutive reuse cycles.^27^ Kholdeeva et al. investigated the stability and catalytic performance of niobium-containing mesoporous silicates in the epoxidation of olefins using hydrogen peroxide.^28^ The findings indicated that the use of methanol as a solvent led to significant leaching of niobium from the catalyst.

Recent research shows that anchoring single-atom niobium (Nb) onto g-C_3_N_4_ significantly improves its photodegradation efficiency.^29^ Incorporating Nb at the pyridinic nitrogen sites significantly improves the separation and directional transfer of photogenerated charge carriers. Consequently, the Nb-modified g-C_3_N_4_ exhibits superior electrical conductivity, a substantially reduced electron–hole recombination rate, and a narrowed band gap that boosts visible-light absorption.^29^ These enhancements contribute significantly to the photodegradation of persistent pollutants, such as amoxicillin, and facilitate the efficient reduction of CO_2_ into valuable products, including CO and CH_4_.^29^

In this work, we report the synthesis of a Nb-doped g-C_3_N_4_ (NbCN) catalyst via a straightforward pyrolysis approach, aiming to develop a highly active and stable system for oxidative desulfurization. Structural characterization reveals that Nb species are highly dispersed and anchored to pyridinic nitrogen sites within the g-C_3_N_4_ framework, forming well-defined active centers. The catalytic performance of the resulting NbCN material was systematically evaluated in the oxidative removal of dibenzothiophene (DBT) using H_2_O_2_ as a green oxidant, with particular emphasis on the effects of key reaction parameters, including temperature, catalyst loading, and O/S molar ratio.

Beyond activity, special attention was given to addressing two major limitations of conventional ODS systems: deactivation and nitrogen poisoning. The catalyst exhibits remarkable stability over multiple reuse cycles with negligible niobium leaching, as well as an unusual tolerance to nitrogen-containing compounds. These combined features highlight the potential of Nb-doped g-C_3_N_4_ as a robust and N-tolerant catalytic platform, offering a promising route toward the practical implementation of ultralow sulfur fuel technologies.

## 2 Experimental Section

### 2.1 Catalyst Preparation

All chemicals were of reagent grade and used without further purification. Pure g-C_3_N_4_ was synthesized following previously reported methods.^19,30^ Briefly, 6.3 g of melamine (50 mmol) was dissolved in 100 mL of hot distilled water (100 °C) with 5 mL of HCl (37%) under magnetic stirring for 30 min. A white precipitate formed upon cooling, which was then dried at 80 °C overnight to remove residual moisture. The resulting solid was placed in a semiclosed alumina crucible and heated at rate of 10 °C/min up to 550 °C for 2 h in a muffle furnace under ambient conditions. After cooling to room temperature, the obtained yellow agglomerates were ground into a fine powder using an agate mortar for further use. Nb-doped g-C_3_N_4_ was prepared following the same procedure used for pure g-C_3_N_4_ by adding ammonium niobate­(V) oxalate hydrate (Sigma-Aldrich) to the solution containing 6.30 g of melamine. Precursor masses of 0.1515, 0.7575, and 1.5149 g were used to prepare the 1-NbCN, 5-NbCN, and 10-NbCN materials, respectively. The 0-NbCN sample was prepared without addition of the Nb precursor.

### 2.2 Characterization Techniques

X-ray powder diffraction (XRD) patterns were recorded using a Rigaku D/MAX 2400 diffractometer equipped with a Cu Kα radiation source (λ = 1.54 Å). Thermogravimetric analysis (TGA) was performed on a Shimadzu DTG60/60H analyzer under synthetic air at a flow rate of 50 mL min^–1^, maintaining a heating rate of 10 °C min^–1^. Fourier transform infrared (FTIR) spectra were collected using a Shimadzu IRAffinity-1 spectrophotometer. Nitrogen adsorption–desorption isotherms were recorded at 77 K using a NOVA 1000e Surface Area and Pore Size Analyzer (Quantachrome Instruments, Boynton Beach, FL, USA). The specific surface area (*S*_BET_) was calculated by the Brunauer–Emmett–Teller method. The micropore surface area (S_micro_) and micropore volume (V_micro_) were determined by the t-plot and αs-plot methods, respectively. The total pore volume (*V*_total_) was estimated from the amount of nitrogen adsorbed at the highest measured relative pressure (P/P_0_ ≈ 1.0). Pore-size distributions were obtained by the Barrett–Joyner–Halenda (BJH) method using the desorption branch. Scanning electron microscopy (SEM) and elemental mapping via energy-dispersive X-ray spectroscopy (EDX) were carried out using an FEI Quanta microscope at accelerating voltages of 15–30 kV. High-resolution transmission electron microscopy (HRTEM) images were obtained with an FEI Tecnai G2 microscope operating at an acceleration voltage of 100 kV. Electron paramagnetic resonance (EPR) measurements were conducted using a Miniscope 400 spectrometer (Magnettech, Germany).

### 2.3 Oxidative Desulfurization

Dibenzothiophene (DBT, Sigma–Aldrich) was dissolved in cyclohexane to prepare a model fuel with a sulfur concentration of 500 ppm. The oxidation reactions were conducted in a 50 mL two-necked flask equipped with a magnetic stirrer and a reflux condenser. A total of 25 mL of the model fuel was added to the reactor, which was maintained at 60 °C in a thermostatically controlled water bath. The oxidative desulfurization experiments were conducted using 25.0 mL of model fuel containing 500 ppm S of DBT and 5.0 mL of acetonitrile, corresponding to an acetonitrile/cyclohexane volume ratio of 1:5. The reactions were performed with a catalyst concentration of 4.0 g L^–1^, O/S = 20, at 60 °C for 120 min. Under the final selected conditions and in the catalyst-reuse experiments, the acetonitrile volume was increased to 25.0 mL, resulting in an acetonitrile/cyclohexane ratio of 1:1, while all other reaction parameters were maintained unchanged. The mixture was stirred vigorously at a constant speed using a magnetic stirrer. The DBT concentration in the cyclohexane phase was monitored at fixed time intervals using a gas chromatograph. GC analysis was also performed on the acetonitrile phase at the end of the reaction to verify the formation of oxidized products. The DBT removal efficiency (%) was determined using the initial DBT concentration (C_0_) and its concentration after t minutes of reaction (C_t_), according to the equation: Removal % = (1 – C_t_/C_0_) × 100%.

The volume and mass of the reaction mixture were assumed to remain constant, as only small liquid samples were withdrawn during analysis. Recycling tests were performed under identical conditions. After each reaction cycle, the catalyst was separated from the mixture via centrifugation and reused directly or after purification by washing several times with ethanol. Comprehensive two-dimensional gas chromatograph system.

The influence of quinoline on DBT oxidative desulfurization was evaluated using the same procedure described above. Quinoline was added to the model fuel at 100, 250, or 500 ppm N, corresponding to N/S ratios of 1:5, 1:2, and 1:1, respectively. The experiments were performed using 25.0 mL of model fuel containing 500 ppm S of DBT, 5.0 mL of acetonitrile, a catalyst concentration of 4.0 g L^–1^, and O/S = 20 calculated relative to DBT, at 60 °C for 120 min under constant magnetic stirring and without an external irradiation source. DBT in the cyclohexane phase was monitored by GC at 0, 10, 20, 30, 60, 90, and 120 min, following the same sampling procedure employed in the standard kinetic experiments. To evaluate possible quinoline oxidation, a separate control experiment was performed with quinoline dissolved exclusively in acetonitrile, without DBT, using O/N = 20 and the same catalyst concentration, temperature, and reaction time.

The oxidative desulfurization performance of 10-NbCN was evaluated using a commercial Brazilian S500 diesel matrix intentionally fortified with 100 ppm S of each sulfur compound—dibenzothiophene, methyl phenyl sulfide, and diphenyl sulfide, corresponding to a nominal total added sulfur concentration of 300 ppm S. This fortification enabled compound-specific monitoring of representative aromatic and thioether sulfur substrates while preserving the compositional complexity of the commercial diesel matrix. The experiment was conducted using 25.0 mL of spiked diesel, 5.0 mL of acetonitrile, a catalyst concentration of 4.0 g L^–1^, and O/S = 20 calculated relative to the total added sulfur, at 60 °C for 120 min. The intentionally added sulfur compounds were monitored individually by GC × GC/MS before and after the reaction. The determinations were performed on a comprehensive two-dimensional gas chromatography system 8890 equipped with a simple single quadrupole mass spectrometer (GC × GC/MS) MSD 5577B from Agilent Technologies (Santa Clara, USA). The system used a DB-5MS capillary column (20.0 m × 0.180 mm × 0.180 μm) as first dimension (1D), and a DB-17HT capillary column (5.0 m × 0.250 mm × 0.150 μm) as second dimension (2D). The chromatographic separation was performed under the following optimized conditions: hydrogen was used as carrier gas with a constant flow rate of 0.5 mL min^–1^; the injector temperature was set at 300.0 °C and operated in split 1:25. The oven temperature program began at 40.0 °C (held for 2.0 min), followed by a linear ramp at 3.0 °C min^–1^ to 280.0 °C (final hold time of 3.0 min). The transfer line temperature was 320.0 °C. The mass spectrometer was operated in electron ionization mode at 70 eV and an ion source temperature of 320 °C, a storage rate of 20 Hz and a mass range of 45–350 u. The chromatographic system incorporated an Agilent G4566A Reverse Fill/Flush CFT GC × GC differential flow modulator. The modulator was precisely optimized with the following parameters: 10.000 s modulation period and 0.400 s injection duration. All ODS experiments were conducted in a thermostatically controlled water bath without an external irradiation source.

### 2.4 Electronic Structure Calculations

Electronic structure calculations were performed using the ORCA 6.0 package.^31,32^ The reported results are based density functional theory (DFT) calculations using the ωB97X-D functional^33,34^ and the def2-SVP basis set.^35^ Three heptazine units, with the terminal bonds passivated by hydrogen, were used to model pristine g-C_3_N_4_. An isolated [NbO]^3+^ fragment coordinated by the pyridinic nitrogen atoms surrounding a g-C_3_N_4_ cavity was adopted as a chemically plausible model for the Nb-containing material. The terminal oxo motif was motivated by the oxoniobium­(V) coordination environment of the ammonium niobium oxalate precursor, commonly represented as NH_4_[NbO­(C_2_O_4_)_2_(H_2_O)_2_]·nH_2_O. Although the molecular precursor decomposes during calcination and is not assumed to retain its original coordination sphere, its oxoniobium­(V) structure provides a reasonable starting point for evaluating the structural plausibility and local stability of an Nb­(V)-oxo species coordinated to the nitrogen-rich g-C3N4 framework. Mayer bond orders^36,37^ were calculated to analyze the strength of interactions specific interactions. The geometry of all structures were optimized without constraints, and harmonic frequencies were obtained to confirm that there was no normal mode that presented imaginary frequencies.

## 3 Results and Discussion

The FTIR spectra for niobium oxide (Nb_2_O_5_), synthesized by calcining ammonium niobium oxalate and Nb-doped g-C_3_N_4_, are presented in Figure 1a. The FTIR spectrum of Nb_2_O_5_ exhibits two broad overlapping bands: a more intense band near 470 cm^–1^ and a less intense one around 800 cm^–1^, in agreement with literature reports.^38,39^ The absorption band at approximately 470 cm^–1^ is attributed to Nb–O stretching (480–540 cm^–1^), while the band near 800 cm^–1^ corresponds to Nb=O stretching, Nb–O–Nb angular vibrations, and asymmetric Nb–O–Nb stretching.^34,36^ For the NbCN materials, the characteristic g-C_3_N_4_ bands appear near 3180 cm^–1^ (N–H stretching), 1200–1700 cm^–1^ (C–N and C=N stretching), and 804 cm^–1^ (triazine ring breathing).^30,41^ Nb (0–10%) does not significantly change the g-C_3_N_4_ structure, as all spectra exhibit identical band positions and relative intensities. This supports the notion of highly dispersed metal species in g-C_3_N_4_.

**Figure 1:**
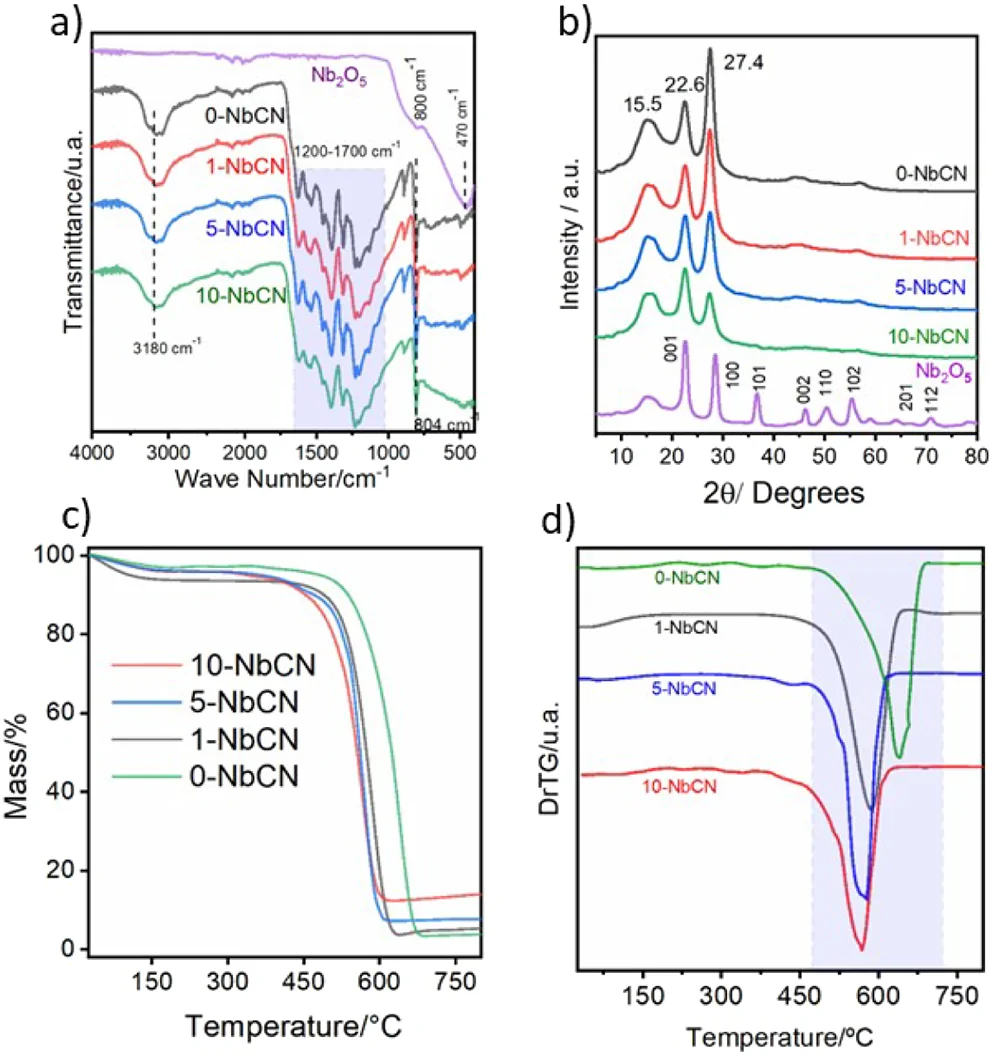
(a) FTIR spectra and (b) XRD patterns of Nb_2_O_5_ and the synthesized NbCN materials; (c) TGA and (d) DTG profiles of g-C_3_N_4_ and the NbCN materials obtained under synthetic air.

Figure 1b presents the X-ray diffraction (XRD) patterns of the synthesized materials. The pure g-C_3_N_4_ (0-NbCN) exhibits a diffraction pattern partially deviating from that of pure g-C_3_N_4_ (JCPDS 87-1526) compared to literature data.^42,43^ Graphitic carbon nitride shows a diffraction peak at 13.1°, indexed to the (100) plane, arising from the repetition pattern of heptazine units, and a more intense peak at 27.4°, attributed to the (002) plane, characteristic of the stacking of aromatic conjugated layers typical of graphitic materials.^43^ The XRD results for all g-C_3_N_4_-derived samples (0–10 NbCN) show a peak at 2θ = 27.4°, corresponding to the (002) plane. The peak’s intensity decreases as the niobium content increases from 0% to 10%, likely due to decreased g-C_3_N_4_ crystallinity resulting from niobium incorporation, consistent with prior studies.^29,30^ Additionally, a broad peak of equal relative intensity is observed at 2θ = 15.5°, slightly shifted from the expected position for the (100) plane at 13.1°, present in all C_3_N_4_-derived materials. Furthermore, all samples exhibited a peak at 2θ = 22.6°, which is atypical for g-C_3_N_4_-derived materials. This peak may be associated with the formation of melem-derived intermediates resulting from incomplete polycondensation of melamine.^44,45^ Furthermore, no diffraction peaks attributed to niobium phases are observed in the XRD spectra of the NbCN samples, suggesting that the Nb­(V) ions are well-dispersed within the g-C_3_N_4_ structure.^40^.

Thermogravimetric analysis (TGA) and derivative thermogravimetry (DTG) curves (Figure 1c,d) reveal the mass loss profiles of g-C_3_N_4_, 1-NbCN, 5-NbCN, and 10-NbCN under synthetic air flow. All materials exhibited similar behavior, with minor mass losses (<300 °C) attributed to adsorbed water/impurities and a major decomposition step between 450–650 °C.^46^ Pure g-C_3_N_4_ decomposed completely between 540–700 °C (maximum rate at 640 °C), leaving a 3% residue. In contrast, Nb/CN hybrids showed lower thermal stability, with maximum decomposition rates at 585 °C (1-NbCN), 577 °C (5-NbCN), and 565 °C (10-NbCN). This reduced stability likely stems from structural defects induced by Nb incorporation and/or catalytic oxidation of g-C_3_N_4_ by Nb_2_O_5_. Residual masses of 5.2%, 7.5%, and 13.2% were observed for 1-NbCN, 5-NbCN, and 10-NbCN, respectively, ascribed to Nb_2_O_5_ formation. Corresponding Nb content was estimated at 1.5%, 3.1%, and 7.1%, confirming successful Nb integration. The textural properties of the NbCN samples were additionally evaluated by N_2_ adsorption–desorption measurements (Figure S1 and Table S1). The *S*_BET_ values of 0-NbCN, 1-NbCN, 5-NbCN, and 10-NbCN were 26.9, 48.4, 30.8, and 53.8 m^2^ g^–1^, respectively, while their total pore volumes were 0.12, 0.21, 0.14, and 0.18 cm^3^ g^–1^. The comparatively small micropore surface areas and volumes indicate that microporosity makes only a minor contribution to the overall texture of these materials. The pronounced increase in N_2_ uptake at high relative pressures is consistent with the presence of larger pores and interparticle voids, while the BJH profiles of 0-NbCN and 10-NbCN show pore-width contributions around 1.9 and 7.5 nm (Figure S1b). Nevertheless, compared with pristine 0-NbCN, 10-NbCN exhibited an approximately 2-fold increase in *S*_BET_ and ca. 50% increase in total pore volume, demonstrating that Nb incorporation increased the accessible surface area and pore volume of this material.

SEM images (Figure 2) reveal distinct morphologies for g-C_3_N_4_, 5-NbCN, and 10-NbCN. Pure g-C_3_N4 (Figure 2a) exhibits a porous, irregular surface with cavities, typical of NH_3_ gas bubbles formed during melamine condensation.^47^ This porosity enhances surface area and active-site availability, critical for heterogeneous catalysis. The 5-NbCN hybrid (Figure 2b) retains 3D ultrathin g-C_3_N_4_ nanosheets with folded edges, indicating structural preservation post 5% of Nb incorporation. In contrast, 10-NbCN displays dual morphologies: rod-like structures (∼1 μm wide, 5–10 μm long, Figure 2c) and a porous, g-C_3_N_4_-like irregular matrix (Figure 2d). These results suggest that Nb ions influence melamine polymerization, likely occupying pyridinic sites and disrupting the π-conjugated network, thereby promoting anisotropic growth, as observed in analogous metal-doped systems.^48,49^ Figure 2e displays Transmission Electron Microscopy (TEM) images of the 10-NbCN material, revealing a grain with a distinct layered and folded-sheet-like morphology, characteristic of graphitic carbon nitride (g-C_3_N_4_). This structure is indicative of the material’s two-dimensional (2D) architecture, where layers of triazine or heptazine units are weakly bonded by van der Waals forces, leading to the observed folding and stacking patterns. Such features are consistent with the structural properties of g-C_3_N_4_, as reported in similar studies on layered carbon-based materials. The transmission electron microscopy (TEM) images of the prepared g-C_3_N_4_ (Figure 2e) and 10-NbCN (Figure 2f) materials revealed that both samples exhibit layered, platelet-like morphologies on a micrometer scale. This structural feature is characteristic of graphitic carbon nitride (g-C_3_N_4_), confirming the preservation of its two-dimensional (2D) architecture even after Nb incorporation. Notably, no segregated NbOx nanoparticles were observed within the spatial resolution of conventional TEM. Together with the absence of detectable crystalline NbOx reflections in the XRD patterns and the preservation of the principal g-C3N4 vibrational features in the FTIR spectra, this observation is consistent with highly dispersed Nb-containing species. These findings therefore support a high degree of Nb dispersion. However, definitive identification of the Nb species as isolated atoms and determination of their local coordination environment, including the proposed Nb–N interactions, require further confirmation using direct local-structure techniques, particularly aberration-corrected HAADF-STEM and Nb K-edge XANES/EXAFS.

**Figure 2:**
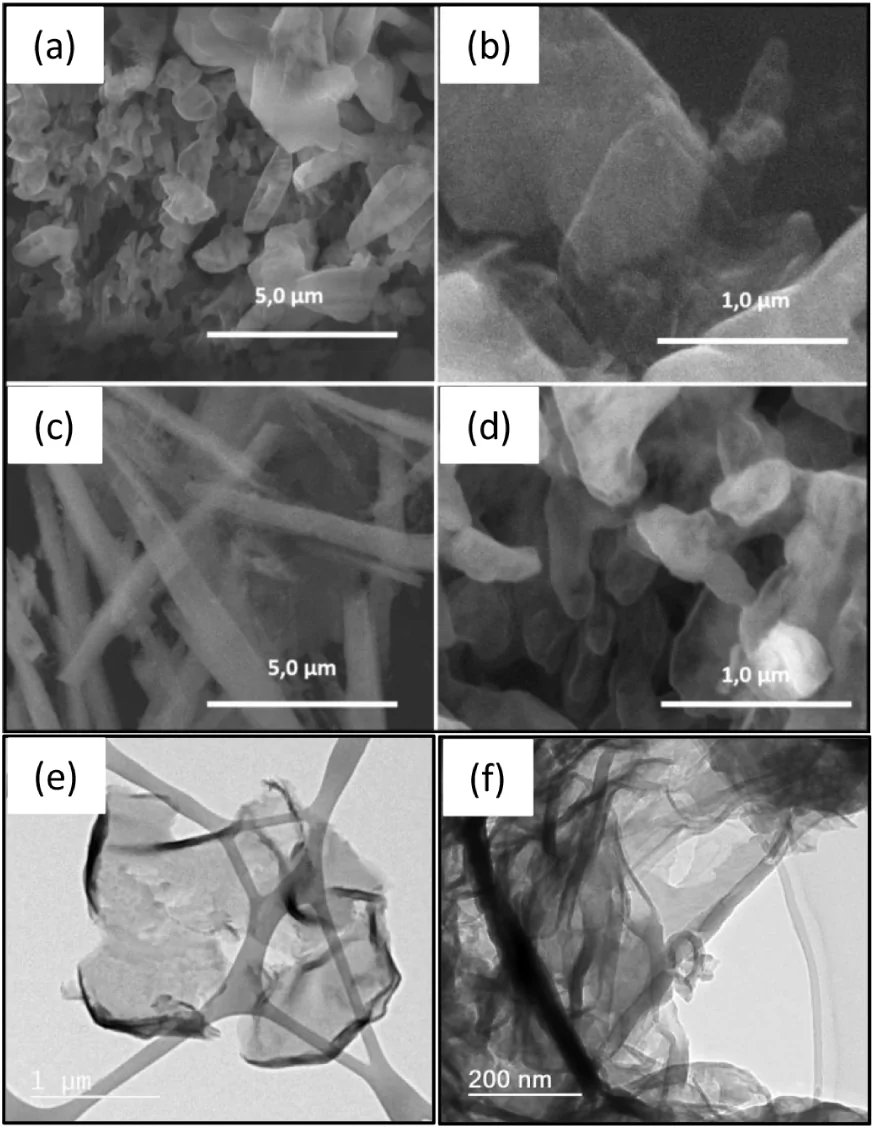
SEM Image of (a) g-C_3_N_4_, (b) 5-NbCN, (c, d) 10-NbCN. TEM Image of (e) g-C_3_N_4_ and (f) 10-NbCN.

Figure 3a displays the desulfurization kinetics of the synthesized materials. All catalysts exhibited an initial 20% reduction in dibenzothiophene (DBT) concentration within the first 10 min, attributed to compound partitioning between the polar (acetonitrile) and nonpolar (cyclohexane) phases. The pristine g-C_3_N_4_ (0-NbCN) showed no significant catalytic activity, with the observed fluctuations in DBT concentration likely associated with adsorption–desorption equilibrium. Among the Nb-doped samples, 1-NbCN displayed low efficiency, achieving only 30% DBT removal after 120 min of reaction, indicating that the low niobium content results in an insufficient density of accessible active sites. In contrast, increasing the Nb loading led to a marked enhancement in catalytic performance. A progressive improvement in DBT removal was observed for 5-NbCN and 10-NbCN, which achieved approximately 60% and 70% removal after 120 min, respectively. This trend clearly indicates that higher Nb incorporation promotes the formation of a greater number of catalytically active Nb sites, thereby enhancing the overall desulfurization efficiency. For comparison, bulk Nb_2_O_5_, obtained by calcination of ammonium niobium oxalate at 550 °C, exhibited relatively low activity, reaching only 37% DBT removal after 120 min. This inferior performance can be attributed to its lower surface area and reduced exposure of active Nb sites, which limits its catalytic effectiveness. Control experiments showed only 21–23% apparent DBT removal by 10-NbCN without H_2_O_2_ and 18–20% with H_2_O_2_ without catalyst, the latter being comparable to extraction with acetonitrile alone. Thus, neither adsorption nor direct oxidation by H_2_O_2_ accounts for the progressive DBT removal observed with the complete 10-NbCN/H_2_O_2_ system.

**Figure 3:**
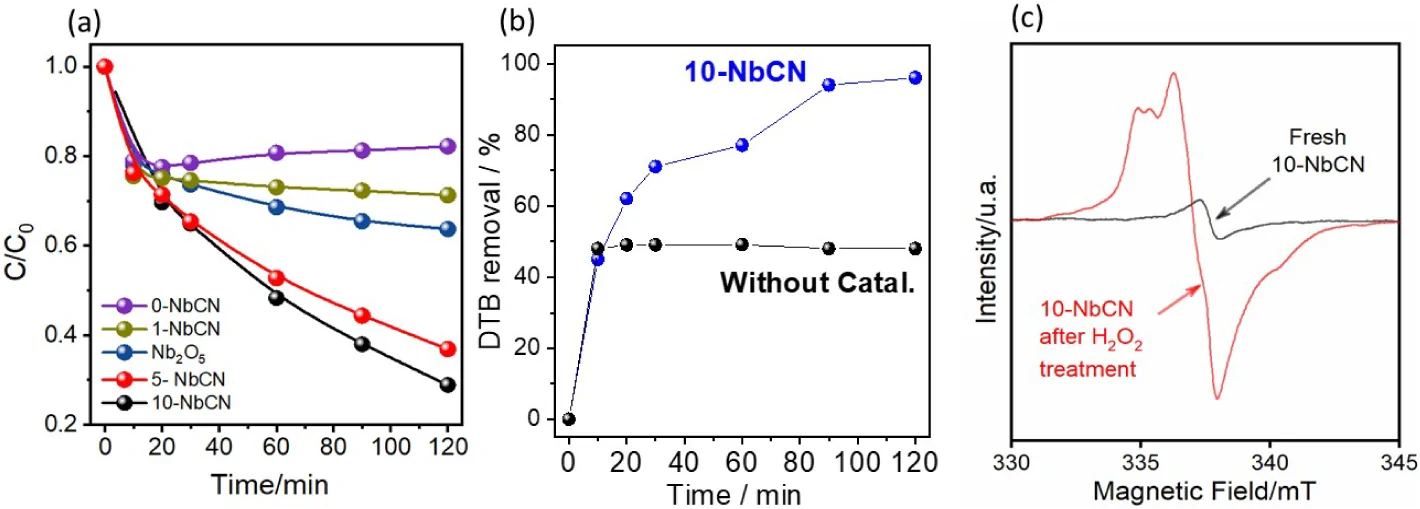
(a) DBT removal kinetics over 0-NbCN, 1-NbCN, 5-NbCN, 10-NbCN, and Nb_2_O_5_ using an acetonitrile/cyclohexane volume ratio of 1:5. (b) DBT removal over 10-NbCN using an acetonitrile/cyclohexane volume ratio of 1:1 and the corresponding control performed with H_2_O_2_ in the absence of catalyst. (c) EPR spectra of 10-NbCN before and after contact with H_2_O_2_. ODS conditions: [S]_0_ = 500 ppm S, O/S = 20, *T* = 60 °C, and catalyst concentration = 4.0 g L^–1^ when applicable.

To further evaluate the performance of the highest-performing composition among the investigated samples, the oxidative desulfurization reaction was performed under optimized extraction conditions by increasing the acetonitrile/cyclohexane volume ratio from 1:5 to 1:1 while maintaining the other reaction parameters unchanged. The kinetic behavior under the original 1:5 condition is presented in Figure 3a, whereas Figure 3b shows the reaction under the selected 1:1 phase ratio together with the control performed with H_2_O_2_ in the absence of catalyst. Under the 1:1 condition, the complete 10-NbCN/H_2_O_2_ system achieved approximately 94% DBT removal after 90 min and 96% after 120 min. The control without catalyst reached approximately 48% apparent DBT removal within the first 10 min and remained nearly constant at 48–49% thereafter, indicating that this initial decrease was predominantly associated with DBT partitioning into the acetonitrile phase. In contrast, DBT removal in the complete catalytic system increased progressively from 45% at 10 min to 96% at 120 min, demonstrating the contribution of Nb-mediated H_2_O_2_ activation and DBT oxidation. A higher acetonitrile/cyclohexane volume ratio increases the fraction and total amount of DBT transferred into the polar reaction phase, improving its availability to the H_2_O_2_-activated catalytic sites and reducing phase-transfer limitations. Therefore, the improvement observed under the 1:1 condition reflects the enhanced overall efficiency of the coupled extraction–oxidation process rather than a change in the intrinsic nature of the 10-NbCN active sites.

The comprehensive two-dimensional chromatograms shown in Figure S2 reveal the composition of the cyclohexane phase before reaction (Figure S2a), the cyclohexane phase after reaction (Figure S2b), and the acetonitrile phase after reaction (Figure S2c). During the course of the reaction, the chromatographic monitoring evidenced the progressive consumption of dibenzothiophene (DBT) accompanied by the transient formation of dibenzothiophene sulfoxide (DBTO), followed by its further oxidation to dibenzothiophene sulfone (DBTO_2_). At the end of the reaction, the cyclohexane phase exhibited a marked decrease in the DBT signal and no detectable chromatographic signals associated with oxidized sulfur species, including DBTO or DBTO_2_. Conversely, the acetonitrile phase contained only a small residual amount of DBT together with a well-defined DBTO_2_ peak, while DBTO was no longer detected. These results indicate that DBTO acts as an intermediate species that is subsequently converted into DBTO_2_, which accumulates in the polar phase due to its higher polarity. Therefore, the chromatographic data provide direct experimental evidence that sulfur removal proceeds through sequential catalytic oxidation of DBT to DBTO and DBTO_2_, followed by selective extraction of the final oxidation product into the acetonitrile phase.

No external irradiation source was employed in the ODS experiments. Consequently, the observed activity is not interpreted as a photocatalytic response of g-C_3_N_4_ or as involving photogenerated charge carriers, but in terms of H_2_O_2_ activation by the Nb-containing catalytic sites. Enhanced catalytic activity in Nb-doped g-C_3_N_4_ can be attributed to its remarkable ability to activate H_2_O_2_ and generate highly reactive peroxo species (Nb–O_2_^•–^). The EPR spectrum of 10-NbCN (Figure 3c) shows a signal at 337 mT that, in the pristine state, originates from unpaired electrons in the aromatic carbon framework of g-C_3_N_4_.^50,51^ Upon H_2_O_2_ treatment, the EPR spectrum of 10-NbCN shows an intense signal at 337 mT with distinct peak positions that can be attributed to the formation of superoxide radical (O_2_^–•^) stabilized at Nb­(V) sites, similar to what occurs with other high valence transition metals with d^0^ configuration such as Ti­(IV), V­(V), W­(VI), Mo­(VI).^30,52–54^ This change suggests that Nb sites function as catalytic centers, facilitating electron-transfer processes that convert H_2_O_2_ into peroxo-intermediates. These peroxo species are especially effective in oxidizing sulfur-containing substrates like dibenzothiophene (DBT), converting them into sulfones or sulfoxides.^52^

The effect of the 10-NbCN dosage and O/S molar ratio on the kinetics of DBT removal was assessed. It was observed that the ODS performance improved with an increase in catalyst dosage from 2 to 8 g L^–1^ (Figure 4a). Following 120 min of reaction, the sulfur removal achieved was 54%, 72%, and 90% for catalyst quantities of 2, 4, and 8 g L^–1^, respectively.

**Figure 4:**
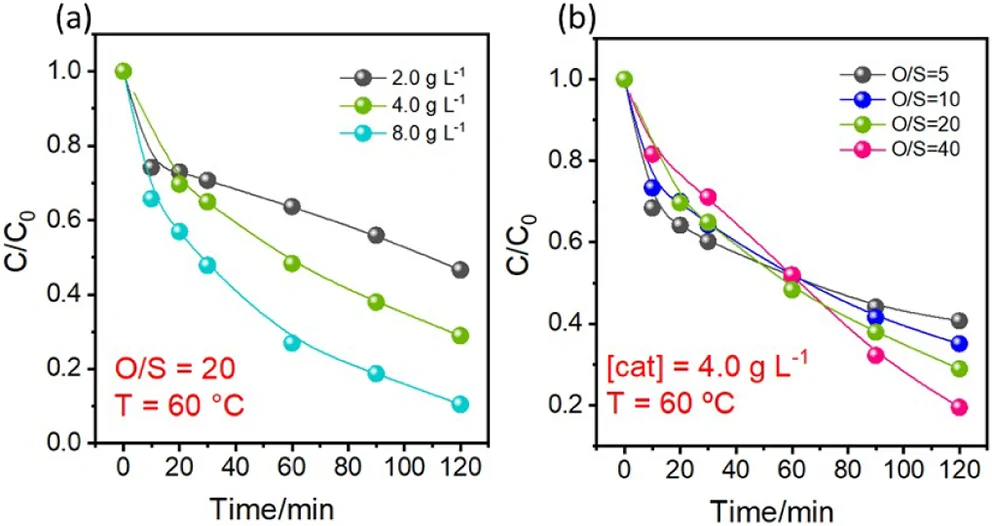
Influence of (a) catalyst dosage and (b) O/S ratio on the kinetics of DBT removal utilizing the 10-NbCN catalyst. Condition: [S]_o_= 500 ppm S, *T* = 60 °C.

The effect of H_2_O_2_ concentration, expressed as the H_2_O_2_/DBT molar ratio, on DBT removal was investigated (Figure 4). As expected, increasing the oxidant concentration leads to a higher DBT oxidation rate, enhancing sulfur removal. Under fixed conditions (60 °C, [cat] = 4 g L^–1^), DBT removal efficiencies of 58%, 63%, 70%, and 81% were achieved for O/S ratios of 5, 10, 20, and 40, respectively, after 120 min reaction. Interestingly, lower O/S ratios produced a faster apparent decrease in DBT concentration during the initial reaction period, whereas higher O/S ratios afforded greater removal at longer reaction times. Because DBT was quantified in the cyclohexane phase, its initial decrease reflects both partitioning into acetonitrile and catalytic oxidation. Increasing the O/S ratio through the addition of aqueous H_2_O_2_ simultaneously increases the water content of the polar phase, which can reduce the extraction capacity of acetonitrile and modify interfacial mass transfer.^55–57^ Therefore, the faster initial decrease at lower O/S ratios is plausibly attributed to more favorable DBT partitioning at the lower water content. At longer reaction times, the greater availability of oxidant becomes predominant, resulting in higher final DBT removal at higher O/S ratios. Since DBT was not monitored simultaneously in both liquid phases, the individual contributions of partitioning, mass transfer, and oxidation cannot be quantitatively separated.

The effects of temperature on the catalytic removal of DBT by the 10-NbCN catalyst were then tested. Maintaining the O/S molar ratio and catalyst concentration fixed, it is evident that desulfurization efficiency increases as temperature rises (Figure 5a) since this directly affects the kinetic rate constant (k). Furthermore, the DBT oxidation rates presented clear pseudo-first-order kinetics (Figure 5b). The apparent rate constants (k) at various temperatures were calculated from the slope of the straight line. The plot of ln­(k) as a function of 1/T yields an apparent activation energy of 41.2 kJ mol^–1^ (Figure 5c), which falls within the range of other values reported in the literature, between 32 and 54 kJ mol^–1^.^58,59^

**Figure 5:**
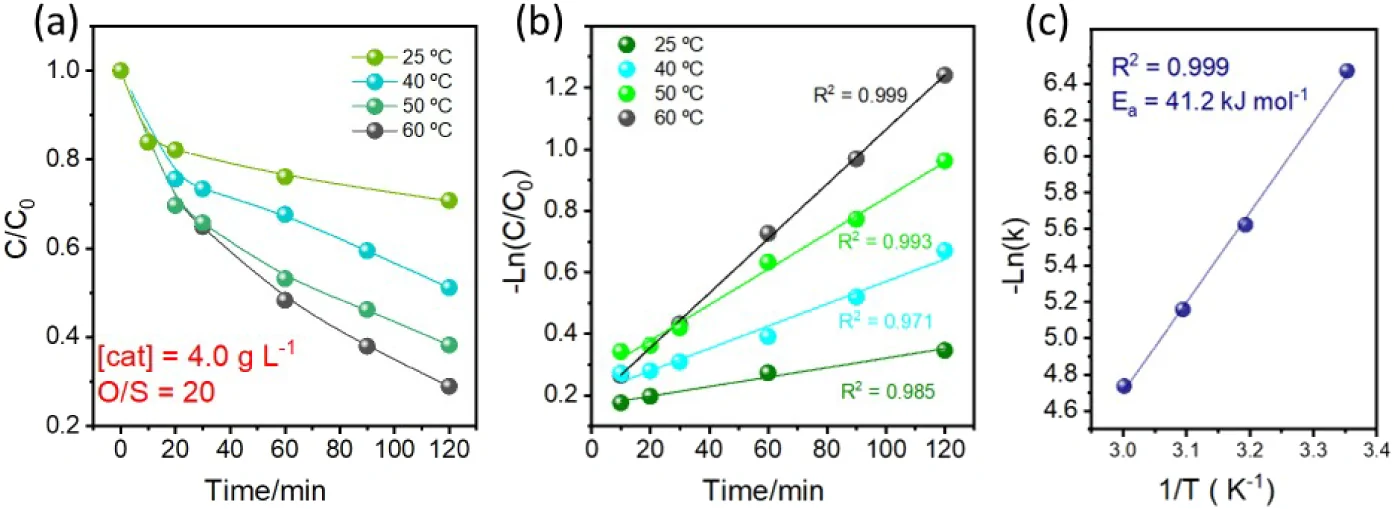
(a) Effect of temperature on the kinetics of DBT removal, (b) the respective pseudo-first-order kinetics, and (c) Arrhenius plot for 10-NbCN catalyst. Conditions: [cat] = 4.0 g L^–1^ [S]_o_= 500 ppm S, O/S = 20.

Nitrogen-containing compounds, such as pyridine, quinoline, indole, carbazole, and acridine, commonly inhibit oxidative desulfurization by competing with sulfur compounds for the oxidant or by strongly interacting with catalytic sites. Figure 6 shows the influence of quinoline on DBT removal at N/S ratios of 1:5, 1:2, and 1:1, corresponding to 100, 250, and 500 ppm N, respectively. In contrast to the inhibitory behavior commonly reported for quinoline, its addition increased DBT removal relative to the nitrogen-free system, which reached approximately 70% after 120 min. The kinetic profiles obtained at N/S ratios of 1:5 and 1:2 were nearly overlapping and resulted in approximately 83% DBT removal, whereas approximately 90% removal was achieved at N/S = 1:1 (Figure 6). These results demonstrate that 10-NbCN is not poisoned by quinoline under the investigated conditions and instead exhibits an unusual promotional effect.

**Figure 6:**
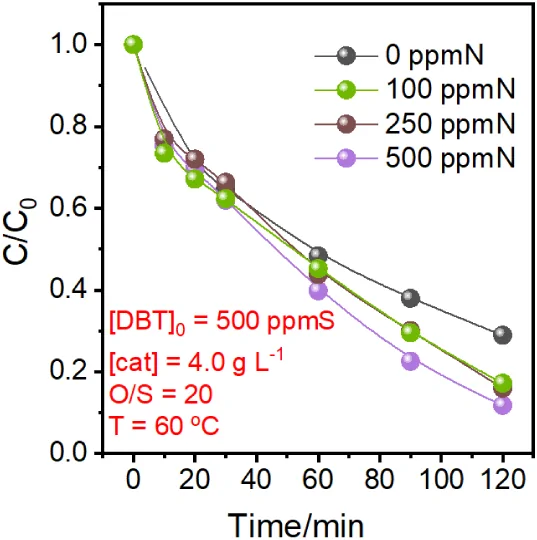
Effect of N-compounds (quinoline) on the kinetics of DBT removal by 10-NbCN. [cat] = 4.0 g L^–1^, [S]_o_ = 500 ppm S, O/S = 20, *T* = 60 °C.

To determine whether this effect involved competitive quinoline oxidation, an additional control experiment was performed under otherwise identical conditions using quinoline in the absence of DBT, with O/N = 20. No appreciable quinoline conversion was detected after 120 min, indicating that quinoline was not significantly consumed as a competing substrate. Therefore, the improvement in DBT removal cannot be attributed to simultaneous quinoline oxidation and is more likely associated with the interaction of quinoline with the catalytic system.

A promotional effect of nitrogen-containing compounds has also been observed in a limited number of catalytic systems. Zhang et al.^60^ reported an enhancement of DBT oxidation, particularly during the initial reaction period, and associated this behavior with the basic character of quinoline and its influence on H_2_O_2_ activation. In a different metal-based system, Liu et al.^61^ showed that coordination of an additional nitrogen-donor ligand to an Fe center can favor the formation of reactive Fe­(IV)=O species. Although this Fe-based mechanism cannot be directly transferred to the present Nb catalyst, it demonstrates that coordination of a nitrogen base does not necessarily result in irreversible catalyst poisoning. In the present system, two nonexclusive effects may contribute to the observed promotion: a transient interaction of quinoline with the Nb/H_2_O_2_ catalytic environment, potentially influencing proton transfer or the formation and reactivity of the proposed Nb–peroxo intermediate, and modification of DBT partitioning or interfacial mass transfer in the biphasic medium. The absence of quinoline conversion supports a catalytic or interfacial role rather than its participation as an oxidizable substrate. Nevertheless, distinguishing these contributions would require in situ spectroscopic and phase-partition measurements, and the exact origin of the promotional effect remains to be fully elucidated.

Figure 7 presents the GC × GC contour plots of the real diesel sample before (Figure 1a) and after 120 min of oxidative desulfurization using the 10-NbCN catalyst (Figure 7b). A comparative analysis of the chromatograms reveals that the overall distribution of hydrocarbons, including saturated and aromatic fractions, as well as nitrogen-containing compounds (e.g., quinolines, pyridines, pyrroles, indoles, and carbazoles), remains essentially unchanged after the reaction. This indicates that the catalytic system does not significantly affect the bulk composition of the fuel. In contrast, a marked reduction in the signals associated with sulfur-containing compounds is observed after the ODS treatment, confirming the high selectivity of the process. This selective removal is a key requirement for practical applications, as it ensures that sulfur species can be efficiently targeted without compromising the integrity of the hydrocarbon matrix, which is directly related to the fuel’s performance. A more detailed evaluation of the individual sulfur compounds shows that methyl phenyl sulfide and diphenyl sulfide were completely removed after 120 min of reaction, as their signals are no longer detected in the chromatogram. On the other hand, dibenzothiophene (DBT) exhibited only partial conversion ca. 55%, indicating a lower reactivity compared to the other sulfur species (Figure S3). Overall, the GC × GC analysis provides strong evidence of the high efficiency and selectivity of the 10-NbCN catalyst, confirming its capability to promote substantial sulfur reduction in complex diesel matrices without compromising the fuel composition.

**Figure 7:**
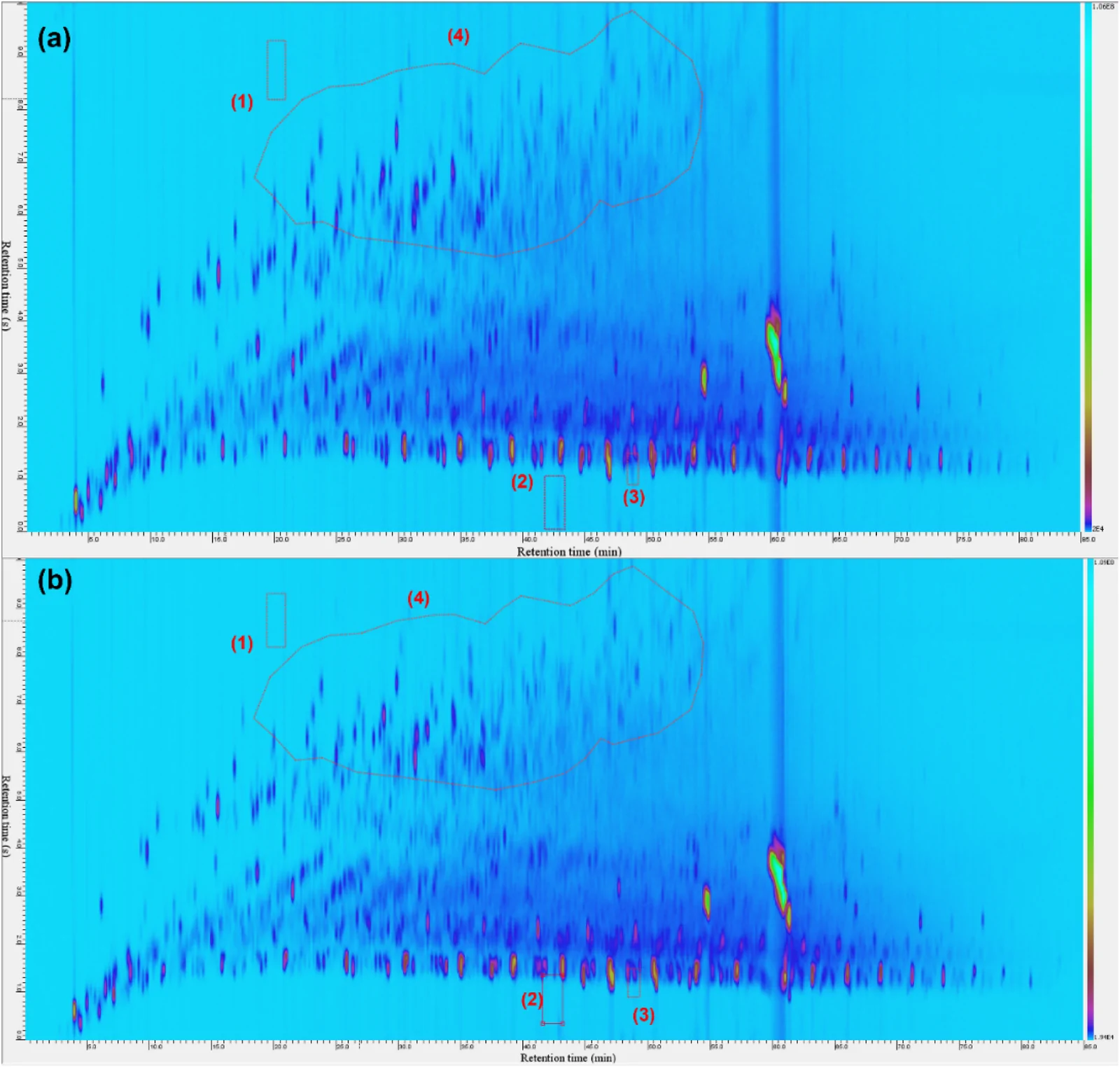
GC × GC color contour plots of diesel (a) before and (b) after oxidative desulfurization with 10-NbCN catalyst. Sulfur compounds: (1) Methyl phenyl sulfide. (2) Dibenzothiophene. (3) Diphenylsulfide; and (4) Nitrogen Compounds.

The stability of the 10-NbCN catalyst was systematically assessed through consecutive reusability tests under the optimized reaction conditions (60 °C, acetonitrile/cyclohexane = 1:1, v/v). As depicted in Figure 8a, the catalyst exhibited outstanding stability over six successive DBT oxidation cycles, maintaining consistently high desulfurization efficiencies between 94% and 97%. The negligible variation in catalytic performance demonstrates the excellent durability of the Nb-doped g-C_3_N_4_ catalyst and indicates that the active Nb sites remain fully accessible throughout repeated oxidation cycles, with no evidence of catalyst deactivation.

**Figure 8:**
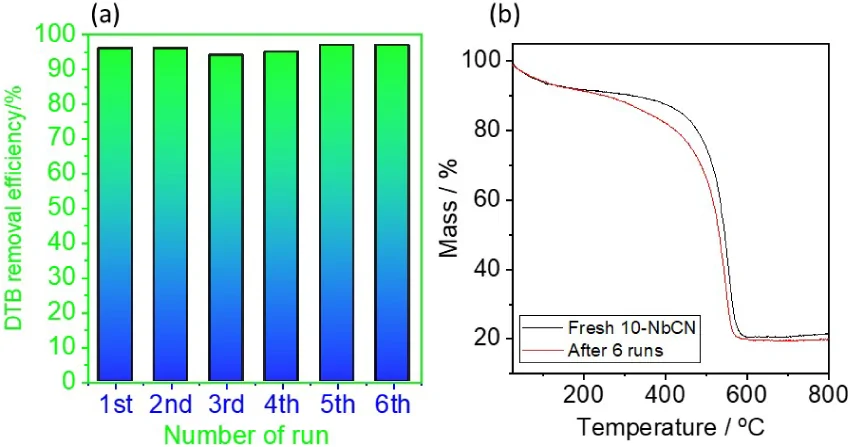
(a) Performance of the recovered catalyst and (b) TGA profiles of fresh and recycled 10-NbCN catalyst.

To further evaluate the structural stability of 10-NbCN, the TGA profiles of the fresh and recycled catalysts were compared (Figure 8b). No appreciable difference in residual mass was observed after six consecutive reaction cycles, indicating preservation of the inorganic content of the material. The FTIR spectra of the fresh and recycled catalysts also remained essentially unchanged, with preservation of the characteristic vibrational bands of the g-C_3_N_4_ framework (Figure S4a). Furthermore, the XRD pattern of the recycled catalyst retained the characteristic diffraction features of fresh 10-NbCN, without the appearance of new reflections attributable to crystalline NbO_*x*_ phases or other segregated crystalline products (Figure S4b). Taken together, the TGA, FTIR, and XRD results indicate that no detectable bulk structural transformation or formation of crystalline Nb-containing phases occurred during the six consecutive oxidative desulfurization cycles, supporting the good structural stability of 10-NbCN under the investigated reaction conditions.

These experimental observations are fully consistent with the theoretical calculations (Figure S5). The DFT-optimized structures indicate that the interaction of the Nb active site with H_2_O_2_ produces only minor changes in the local coordination environment. In particular, the sum of the Mayer bond orders for the Nb–N interactions decreases only slightly, from 2.5 for the pristine Nb site to 2.1 after formation of the NbO_2_ intermediate, while the Nb–O bond lengths remain essentially unchanged (0.23–0.24 nm). These small structural variations indicate that the Nb center remains strongly anchored within the pyridinic cavity of the g-C_3_N_4_ framework throughout the catalytic cycle, preventing Nb migration or leaching even under strongly oxidative conditions. Therefore, the excellent recyclability observed experimentally can be directly correlated with the remarkable structural robustness predicted by the theoretical calculations.

Table S2 places the catalytic behavior of 10-NbCN in the context of representative H_2_O_2_-based ODS catalysts, with emphasis on Nb-containing materials, g-C_3_N_4_-supported systems, recyclability, and the influence of nitrogen-containing compounds.^30,62–67^ The 10-NbCN catalyst combined 96% DBT removal at 60 °C, an apparent activation energy of 41.2 kJ mol^–1^, and essentially unchanged removal efficiency over six consecutive cycles. Hollow Nb_2_O_5_ nanospheres retained high activity over six cycles but exhibited a gradual decrease after the third cycle,^62^ whereas the C_16_–Nb_6_/SiO_2_ polyoxoniobate was reused for 11 cycles with negligible activity loss.^63^ K­[NbO_2_(BDC)] maintained approximately 89–91% DBT removal over five cycles, although at 80 °C and after 180 min,^65^ while Mo/g-C_3_N_4_ decreased to approximately 60% removal in the third cycle.^30^ Thus, 10-NbCN combines high DBT removal under mild thermal conditions, a relatively low apparent activation energy, stable performance over six cycles, and an unusual absence of inhibition by quinoline.

The mechanistic interpretation proposed for DBT oxidation over 10-NbCN is illustrated in Figure 9. Initially, DBT partitions from the cyclohexane phase into the polar acetonitrile phase, where the catalytic oxidation occurs. The EPR spectra show that interaction with H_2_O_2_ changes the paramagnetic environment of 10-NbCN; however, these qualitative spectra do not unequivocally identify a specific reactive oxygen species or directly demonstrate the formation of a closed-shell Nb–peroxo intermediate. Radical-scavenging experiments provided additional mechanistic information. In the presence of 10 mM isopropyl alcohol and 5 mM p-benzoquinone, DBT removals of 94% and 93%, respectively, were obtained after 120 min, compared with approximately 96% without scavengers. Therefore, freely diffusing •OH and O_2_•^–^ are unlikely to be the predominant oxidizing species under the investigated conditions. Similar behavior was reported by Cao et al. for a Ni–MoO_3_/H_2_O_2_ system in decalin/acetonitrile, in which the negligible effects of p-benzoquinone and isopropanol were associated with direct DBT oxidation through a peroxometallic intermediate.^68^

**Figure 9:**
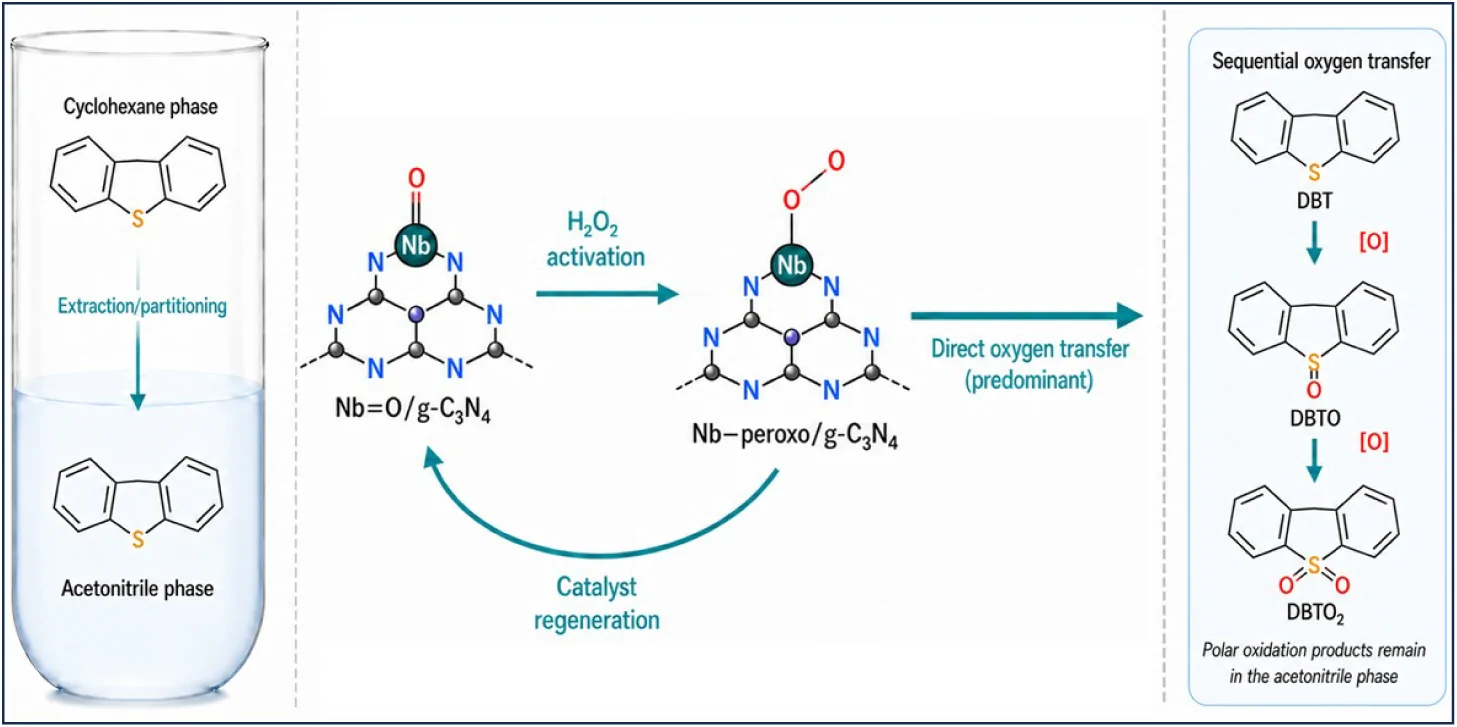
Proposed mechanism for H_2_O_2_-based oxidative desulfurization of DBT over 10-NbCN.

Within the proposed nonradical pathway, the electron-deficient d^0^ Nb­(V) center acts as a Lewis-acidic site for H_2_O_2_ activation. Interaction of H_2_O_2_ with the modeled Nb=O site polarizes the peroxide bond and produces a terminal, angular, and nonbridging Nb–O–O intermediate. The electron-rich sulfur center of DBT then interacts with the electrophilic oxygen of the polarized Nb–O–O group, promoting O–O bond cleavage, S–O bond formation, and regeneration of the Nb=O site. A first oxygen-transfer cycle converts DBT into DBTO, whereas a second cycle converts DBTO into DBTO_2_. The more polar oxidation products remain preferentially in the acetonitrile phase, continuously favoring the coupled extraction–oxidation process. Taken together, the experimental and theoretical results collectively support the proposed catalytic mechanism and demonstrate that the atomically dispersed Nb active centers embedded within the g-C_3_N_4_ framework are responsible for the high catalytic activity, exceptional tolerance toward nitrogen-containing compounds, and excellent stability of the catalyst.

## 4 Conclusion

The Nb-doped g-C_3_N_4_ catalyst (10-NbCN) emerges as a highly efficient and stable material for oxidative desulfurization, achieving near-complete DBT removal (96%) under optimized conditions. Its superior performance stems from Nb integration, which can interact with H_2_O_2_ to produce reactive peroxo species (Nb–O_2_^–^). Remarkably, the catalyst exhibits an unusual tolerance to nitrogen-containing compounds, as quinoline not only fails to inhibit the reaction but promotes DBT conversion even at elevated concentrations. This behavior highlights a distinctive catalytic environment in which Nb active sites preferentially interact with sulfur species, overcoming a key limitation of conventional ODS systems. In addition, the catalyst demonstrates outstanding resistance to deactivation, maintaining consistent activity over six reuse cycles with negligible Nb leaching. This stability is associated with the preservation of Nb–N coordination within the g-C_3_N_4_ matrix under oxidative conditions, preventing metal migration and loss of active sites. This study advances the development of N-tolerant ODS catalysts, offering a sustainable pathway to ultralow-sulfur fuels.

## Supplementary Material



## Notes

N2 adsorption–desorption isotherms and BJH pore-width distributions; textural properties of the NbCN catalysts; GC × GC/MS chromatograms and experimental/library EI mass spectra of DBT, DBTO, and DBTO_2_; GC × GC single-ion chromatograms of spiked commercial Brazilian diesel; FTIR spectra and XRD patterns of fresh and recycled 10-NbCN; DFT-optimized structures and bonding analysis of Nb species before and after interaction with H_2_O_2_; and comparison of 10-NbCN with representative H_2_O_2_-based oxidative desulfurization catalysts (PDF)
