# Supplementary material for: Nb-Doped g‑C3N4: A Highly Efficient and Stable Catalyst for Oxidative Desulfurization
Source: ACS Omega. 2026 Oct 20;11(43):67317–28. doi: 10.1021/acsomega.6c05450 (PMC13737967; doi:10.1021/acsomega.6c05450)
Supplement: Supplementary file 1 [file ao6c05450_si_001.pdf]

Supplementary Information of:

Nb-doped g-C_3N_4: A highly efficient and stable catalyst for oxidative desulfurization

Jennefer V. M. Oliveira[1], Jhonatan B. Oliveira[1], Klaus Krambrock[2], Breno R. L. Galvao[1], Raquel V. Mambrini[1], Eudes Lorençon[1*].

1- Centro Federal de Educação Tecnológica de Minas Gerais (CEFET-MG), Departamento de Química. Av. Amazonas, 5253, Nova Suíça, Belo Horizonte, MG, 30421-169, Brazil.

2- Universidade Federal de Minas Gerais (UFMG), Departamento de Física. Av. Antônio Carlos, 6627, Pampulha, Belo Horizonte, MG, 31270-901, Brazil.

* eudeslorencon@cefetmg.br

Keywords: Oxidative desulfurization, g-C_3N_4-based catalyst, Nitrogen-tolerant catalysis.

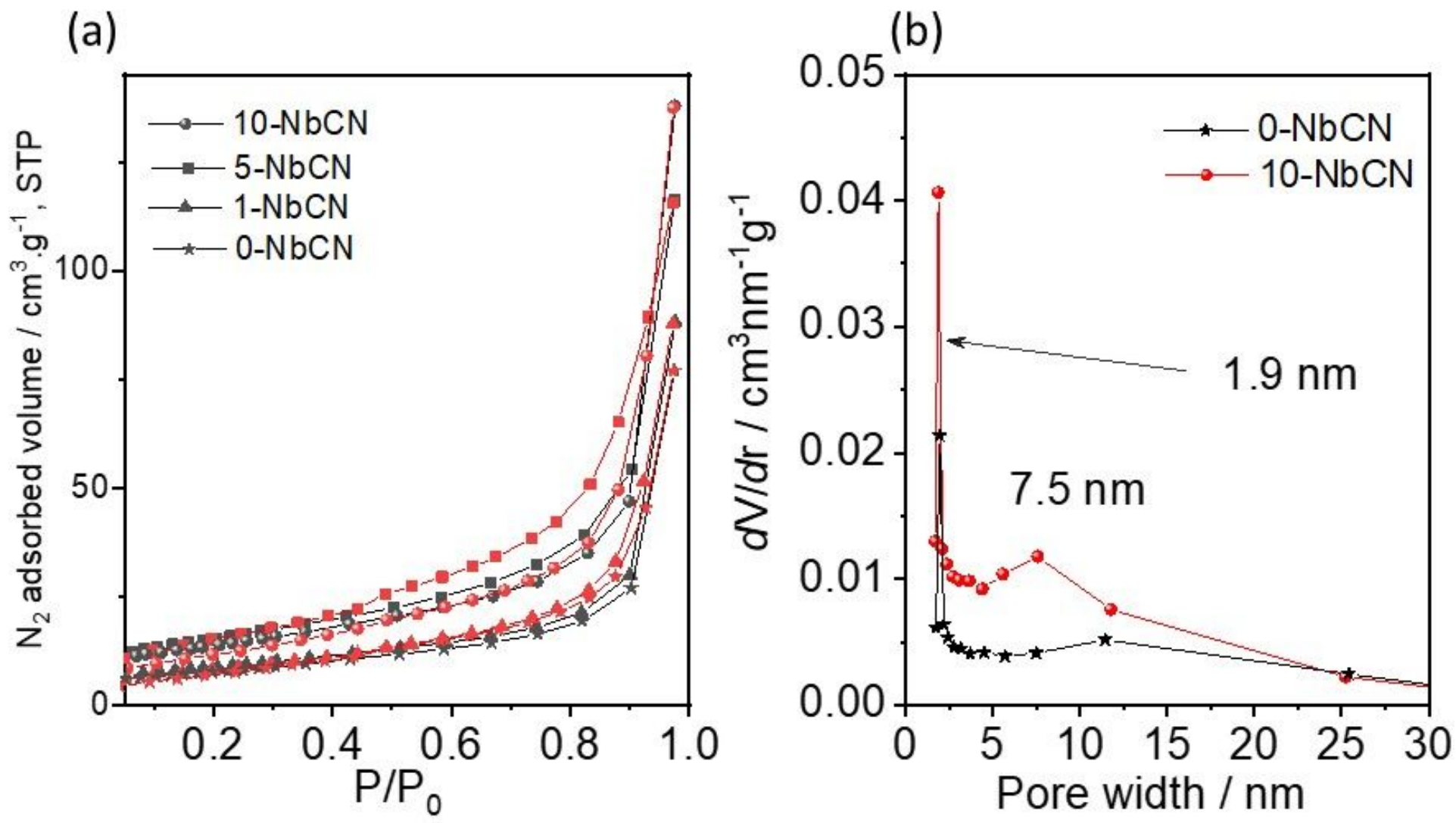

Figure S1. (a) N_2 adsorption-desorption isotherms at 77 K for 0-NbCN, 1-NbCN, 5-NbCN, and 10-NbCN; and (b) BJH pore-width distributions obtained from the desorption branch for 0-NbCN and 10-NbCN.

Table S1. Textural properties of the NbCN catalysts determined from N_2 adsorption-desorption measurements at 77 K.

Catalyst	S_{BET} ($m^2\ g^{-1}$)	S_{micro}[a] ($m^2\ g^{-1}$)	V_{micro}[b] ($cm^3\ g^{-1}$)	V_{total}[c] ($cm^3\ g^{-1}$)
0-NbCN	26.9	5.6	0.002	0.12
1-NbCN	48.4	10.9	0.005	0.21
5-NbCN	30.8	6.1	0.003	0.14
10-NbCN	53.8	6.8	0.003	0.18

[a] Micropore surface area determined by the t-plot method.
[b] Micropore volume determined by the αs-plot method.
[c] Total pore volume estimated from the nitrogen uptake at the highest measured relative pressure ($P/P_0 \approx 1.0$).

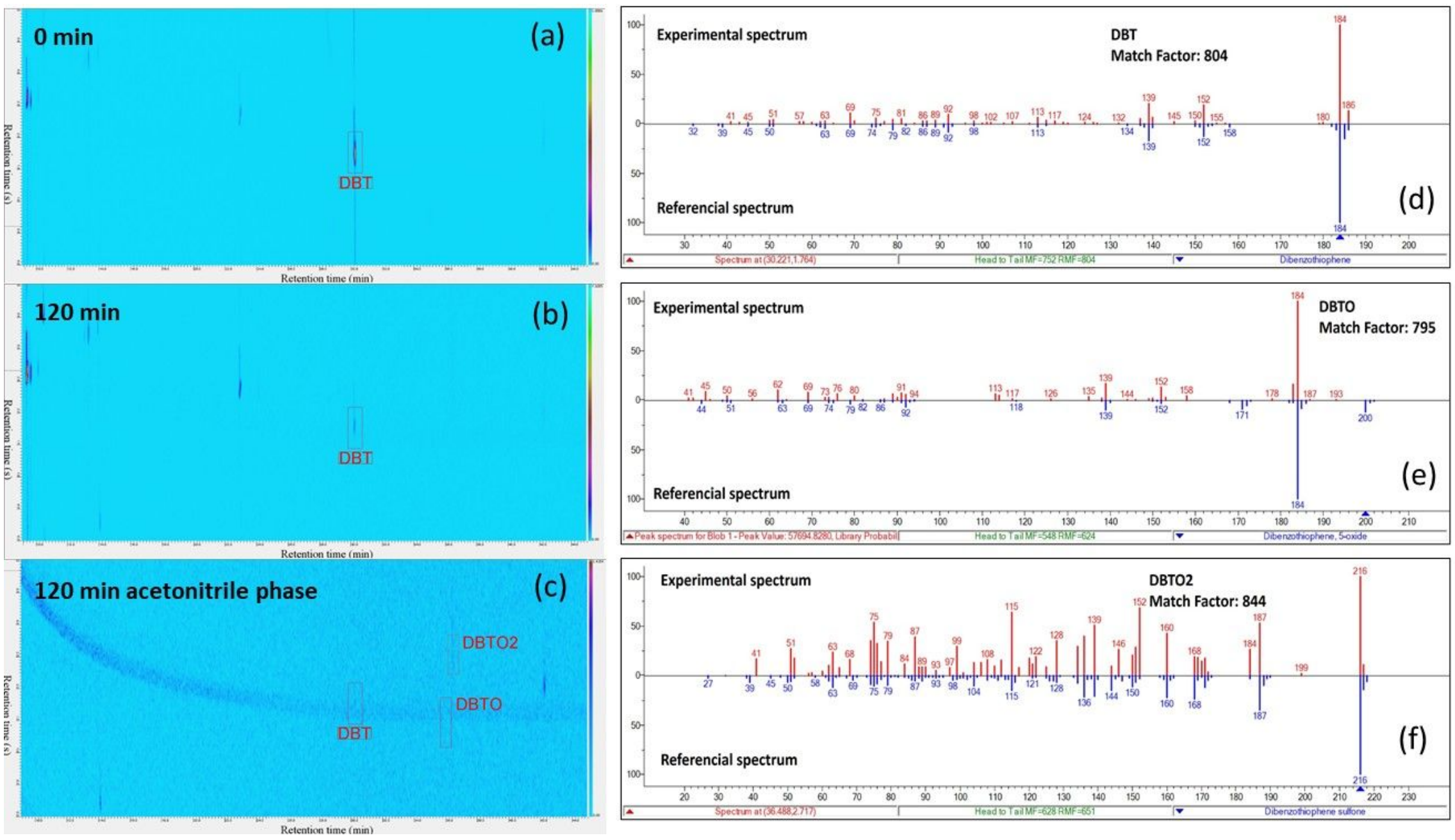

Figure S2. GC×GC/MS characterization of the sulfur-containing compounds during the oxidative desulfurization of DBT over 10-NbCN: (a) cyclohexane phase before reaction, (b) cyclohexane phase after 120 min, (c) acetonitrile phase after 120 min, and head-to-tail comparisons between the experimental EI mass spectra and the corresponding library spectra of (d) DBT, (e) DBTO, and (f) $DBTO_2$. The molecular ions of DBT, DBTO, and $DBTO_2$ were observed at m/z 184, 200, and 216, respectively. Reaction conditions: $[S]_0$ = 500 ppmS, catalyst concentration = 4.0 g L^{-1}, O/S = 20, T = 60 °C, and reaction time = 120 min.

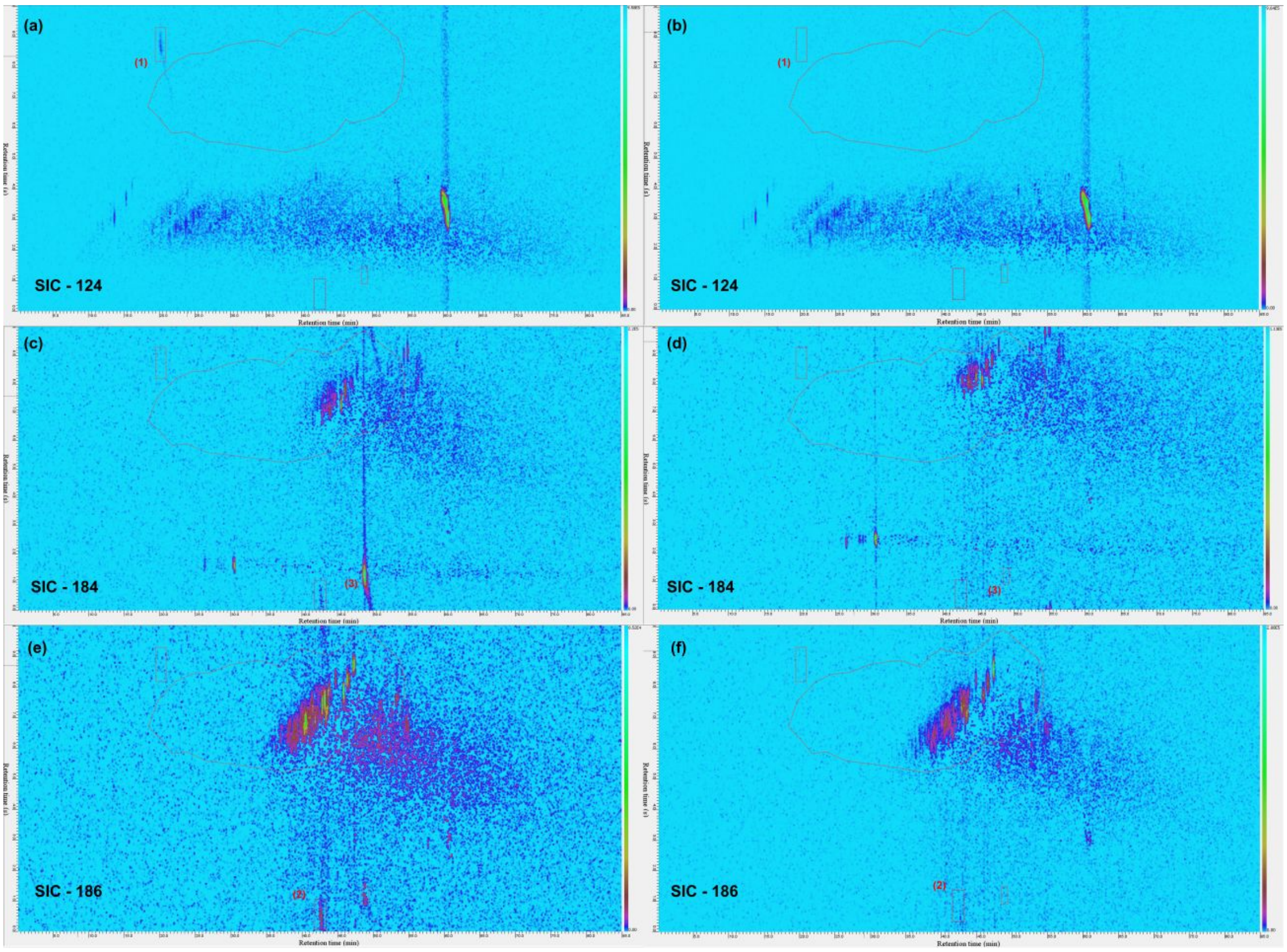

Figure S3. GC×GC single-ion chromatograms of the spiked commercial Brazilian diesel before and after oxidative desulfurization over 10-NbCN: (a,b) m/z 124, methyl phenyl sulfide; (c,d) m/z 184, dibenzothiophene; and (e,f) m/z 186, diphenyl sulfide. The diesel was fortified with 100 ppmS of each sulfur compound. Reaction conditions: 25.0 mL of spiked diesel, 5.0 mL of acetonitrile, catalyst concentration = 4.0 g L^{-1}, O/S = 20 calculated relative to the total added sulfur, T = 60 °C, and reaction time = 120 min.

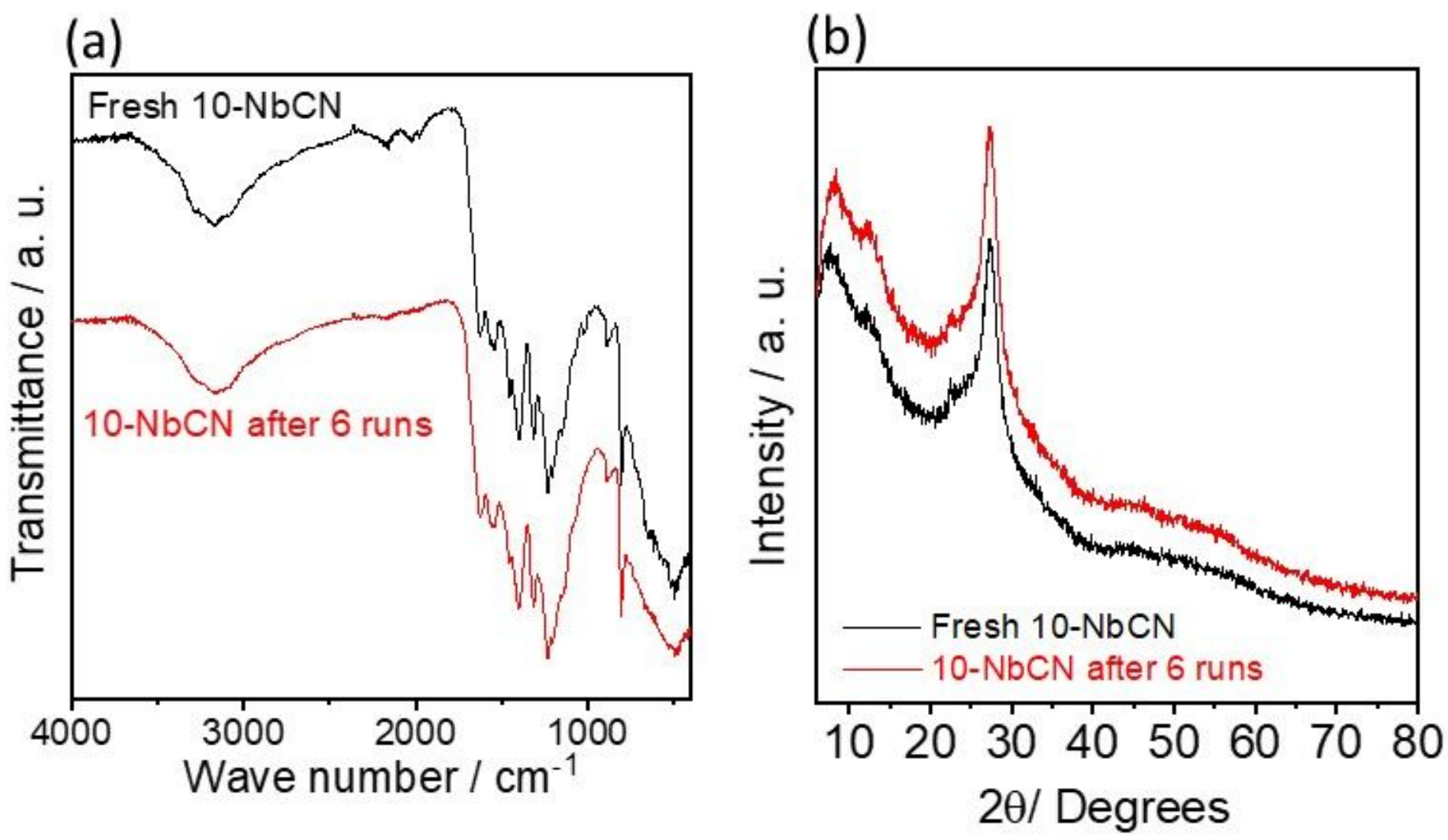

Figure S4. (a) FTIR spectra and (b) XRD patterns of fresh and recycled 10-NbCN after six consecutive oxidative desulfurization cycles.

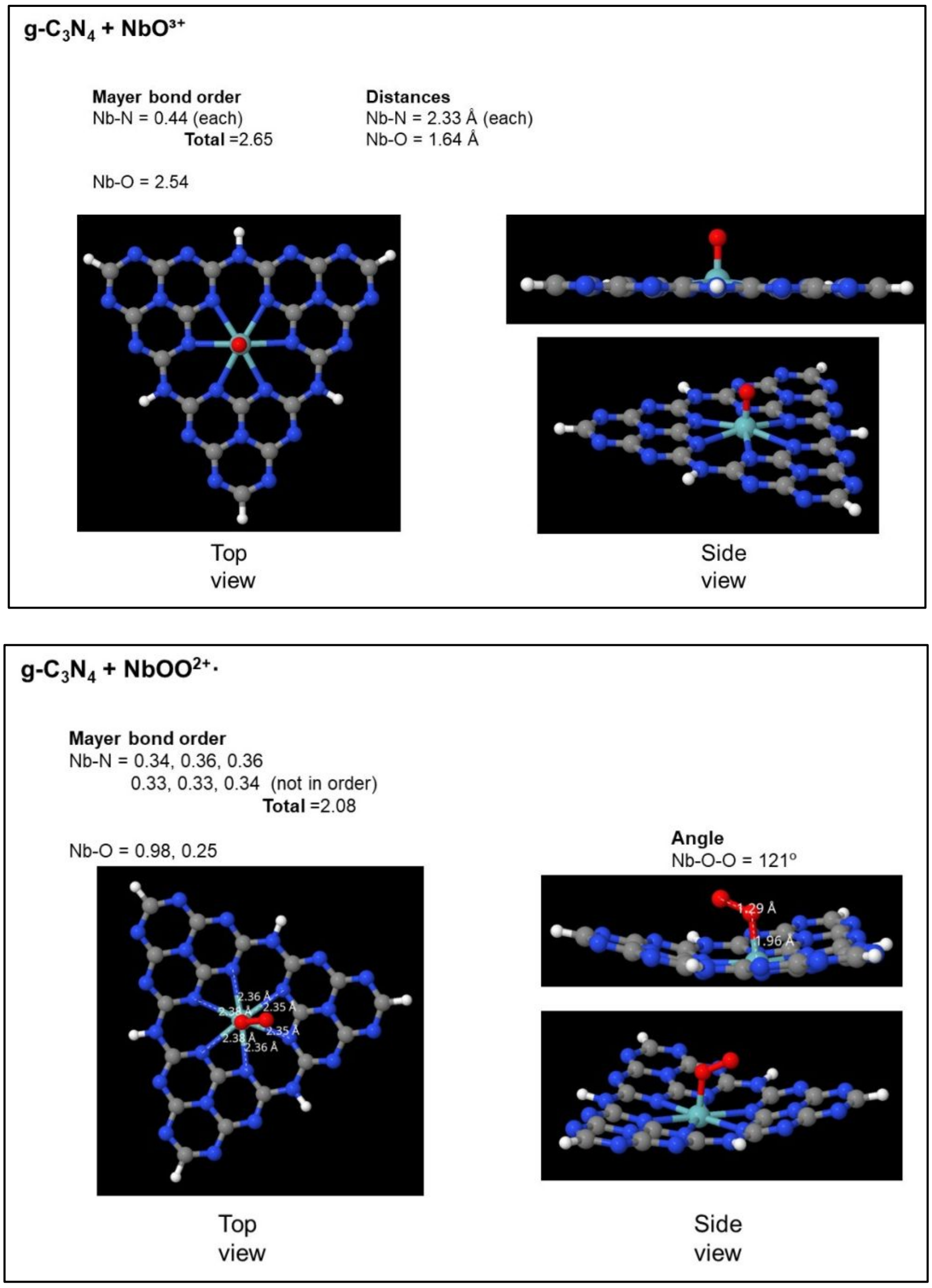

Figure S5. DFT-optimized structures and bonding analysis of Nb species confined in the pyridinic cavity of g-C_3N_4 before and after reaction with H_2O_2.

Table S2. Comparison of 10-NbCN with representative H_2O_2-based catalysts for oxidative desulfurization of dibenzothiophene, emphasizing Nb-containing systems, recyclability, and the effect of nitrogen-containing compounds.

Catalyst	Catalyst loading	Oxidant	O/S	T (°C)	Time (min)	DBT removal/conversion (%)	Ea (kJ mol−1)	Reusability	Effect of nitrogen-containing compounds	Ref. No.
**10-NbCN**	**4.0 g L−1**	**H_2O_2**	**20**	**60**	**120**	**96 [a]**	**41.2**	**Six cycles without significant loss (94–97%)**	**Quinoline promoted DBT removal from approximately 70% to 90% at N/S = 1; no measurable quinoline removal [b]**	**—**
Hollow Nb_2O_5 nanospheres	0.2 g per 500 g model oil	H_2O_2	6	60	60	98.5	N.R.	Six cycles; gradual decline after cycle 3, with 96.9% in cycle 6	N.E.	62
C_{16}-Nb_6/SiO_2 amphiphilic polyoxoniobate	mDBT/mcat = 53.33 mg g−1 [c]	H_2O_2	5	60	40	100	N.R.	Eleven cycles with negligible activity loss	N.E.	63
NbVOx-CA12 mixed oxide	0.125 wt %	H_2O_2	6	60	90	≈90	N.R.	N.R.	N.E.	64
K[NbO_2(BDC)] (MC1)	1.5 g L−1	H_2O_2	≈31 [d]	80	180	90.4	N.R.	Five cycles; 88.5–91.4% removal	Quinoline oxidation was evaluated separately; simultaneous DBT–quinoline behavior was not tested	65
Mo/g-C_3N_4	4.0 g L−1	H_2O_2	20	60	120	≈93	43.6	Activity decreased to approximately 60% in cycle 3	N.E.	30
α-MoO_3 nanobelts (MoNB)	2.0 g L−1	H_2O_2	2.5	60	120	≈100	89.6	Five cycles without significant activity loss	At N/S = 1, DBT removal decreased to approximately 90%, while approximately 80% quinoline was removed	66
NbV/SBA-15 prepared by impregnation	N.R.	H_2O_2	N.R.	60	15	Up to 98	N.R.	N.R.	N.E.	67

Notes: [a] The 96% DBT removal and reuse data for 10-NbCN were obtained using an acetonitrile/cyclohexane volume ratio of 1:1. [b] The quinoline experiment corresponds to the original acetonitrile/cyclohexane ratio of 1:5. [c] Catalyst loading was reported as the DBT-to-catalyst mass ratio. [d] Approximate value calculated from the optimized H_2O_2 volume and the O/S equivalence reported by the authors. N.R. = not reported; N.E. = not evaluated. Nitrogen tolerance was considered only when DBT and the nitrogen compound were simultaneously present. Because the model-fuel composition, initial sulfur concentration, catalyst-loading basis, and extraction conditions differ among the studies, the values are intended to provide literature context and should be interpreted together with the reported experimental conditions.
